# On the Efficiency of Individualized Theta/Beta Ratio Neurofeedback Combined with Forehead EMG Training in ADHD Children

**DOI:** 10.3389/fnhum.2018.00003

**Published:** 2018-01-18

**Authors:** Olga M. Bazanova, Tibor Auer, Elena A. Sapina

**Affiliations:** ^1^Laboratory of Affective, Cognitive and Translational Neuroscience, Department of Experimental, Clinical Neuroscience, Federal State Research Institute of Physiology and Basic Medicine, Novosibirsk, Russia; ^2^Department of Neuroscience, Novosibirsk State University, Novosibirsk, Russia; ^3^Department of Psychology, Royal Holloway University of London, Egham, United Kingdom; ^4^MRC Cognition and Brain Sciences Unit, University of Cambridge, Cambridge, United Kingdom; ^5^Laboratory of Biofeedback Computer System, Research Institute of Molecular Biology and Biophysics, Novosibirsk, Russia; ^6^Department of Psychology, Novosibirsk State University of Economics and Management, Novosibirsk, Russia

**Keywords:** neurofeedback training, ADHD, individual alpha activity, EMG, theta/beta ratio

## Abstract

**Background:** Neurofeedback training (NFT) to decrease the theta/beta ratio (TBR) has been used for treating hyperactivity and impulsivity in attention deficit hyperactivity disorder (ADHD); however, often with low efficiency. Individual variance in EEG profile can confound NFT, because it may lead to influencing non-relevant activity, if ignored. More importantly, it may lead to influencing ADHD-related activities adversely, which may even result in worsening ADHD symptoms. Electromyogenic (EMG) signal resulted from forehead muscles can also explain the low efficiency of the NFT in ADHD from both practical and psychological point-of-view. The first aim of this study was to determine EEG and EMG biomarkers most related to the main ADHD characteristics, such as impulsivity and hyperactivity. The second aim was to confirm our hypothesis that the efficiency of the TBR NFT can be increased by individual adjustment of the frequency bands and simultaneous training on forehead muscle tension.

**Methods:** We recruited 94 children diagnosed with ADHD (ADHD) and 23 healthy controls (HC). All participants were male and aged between six and nine. Impulsivity and attention were assessed with Go/no-Go task and delayed gratification task, respectively; and 19-channel EEG and forehead EMG were recorded. Then, the ADHD group was randomly subdivided into (1) standard, (2) individualized, (3) individualized+EMG, and (4) sham NFT (control) groups. The groups were compared based on TBR and EEG alpha activity, as well as hyperactivity and impulsivity three times: pre-NFT, post-NFT and 6 months after the NFT (follow-up).

**Results:** ADHD children were characterized with decreased individual alpha peak frequency, alpha bandwidth and alpha amplitude suppression magnitude, as well as with increased alpha1/alpha2 (a1/a2) ratio and scalp muscle tension when c (η^2^ ≥ 0.212). All contingent TBR NFT groups exhibited significant NFT-related decrease in TBR not evident in the control group. Moreover, we detected a higher overall alpha activity in the individualized but not in the standard NFT group. Mixed MANOVA considering between-subject factor GROUP and within-subject factor TIME showed that the individualized+EMG group exhibited the highest level of clinical improvement, which was associated with increase in the individual alpha activity at the 6 months follow-up when comparing with the other approaches (post hoc t = 3.456, *p* = 0.011).

**Conclusions:** This study identified various (adjusted) alpha activity metrics as biomarkers with close relationship with ADHD symptoms, and demonstrated that TBR NFT individually adjusted for variances in alpha activity is more successful and clinically more efficient than standard, non-individualized NFT. Moreover, these training effects of the individualized TBR NFT lasted longer when combined with EMG.

## Introduction

It has been already well-described that the main characteristics of attention deficit hyperactivity disorder (AHDH), such as inattention, impulsivity and hyperactivity are associated with a phenomenon so called “slow electroencephalogram (EEG)” (Bazanova and Aftanas, 2005, 2010; Arns et al., 2012; Rudo-Hutt, 2014). This phenomenon includes enhancement of the amplitude of low frequency range and decrease of the amplitude of beta range (Lubar et al., 1995; Magee et al., 2005; Kerson, 2013; Simkin et al., 2016), which is why neurofeedback training (NFT) protocol aiming to decrease the theta/beta ratio (TBR) has been an obvious choice of treatment for children with ADHD (Lubar and Shouse, 1976; Monastra, 2005; Arns et al., 2014). However, the results of this conventional protocol is still not overly convincing, because its efficiency is below 70% (Heinrich et al., 2004; Gevensleben et al., 2009; Cortese et al., 2016). Neurofeedback training in ADHD has been aiming to decrease theta activity since the first demonstration by Lubar and Shouse (1976). Monastra and coworkers have also demonstrated the feasibility of TBR NFT (Monastra et al., 2002); however, they did not find statistically significant correlations between quantitative EEG (QEEG) changes and attention performance. One possible explanation is the individual variance in EEG profile. For example, an immature manifestation of the alpha rhythm, which is the dominant frequency of a child, can be mistakenly considered as high theta activity according to standard frequency ranges. It has been also shown by several studies that 4–8 Hz may represent theta for those with alpha peak around 10 Hz, while it may represent alpha for those with alpha peak around 7–8 Hz. Again, the latter is especially common in children below 10 (Kaiser, 2001; Bazanova and Aftanas, 2005, 2010). Alpha activity plays an important role in cognitive, psychomotor, psycho-emotional, and physiological aspects of human brain functions (see Bazanova and Vernon, 2014 for review). Over the last decade, several studies have emphasized alpha oscillations reflecting the top-down mechanism of neuronal inhibition and neuronal efficiency (Pfurtscheller and Lopes da Silva, 1999; Klimesch et al., 2007; Klimesch, 2012). It has been also shown that children with ADHD exhibit higher theta/alpha ratio apart from higher theta/beta ratio (Clarke et al., 2001, 2002). Due to its spectral proximity to the targeted theta activity, alpha activity is especially prone to be (adversely) influenced by a TBR NFT. If alpha bands are not identified correctly, we might end up accidentally suppressing alpha activity during a TBR NFT; which can, again, explain the low efficiency of the training and the worsening of the symptoms (Kaiser, 2001; Bazanova and Aftanas, 2005, 2010). Alpha wave activity is often discussed as referring to a standard frequency range (e.g., 8–12 Hz) regardless to the oscillatory feature known as the “Berger effect” (Kirschfeld, 2005). The actual alpha activity may or may not be fully represented by this standard range, which may confound findings (Nunez et al., 2001). Luckily, individual theta and beta ranges can be identified based on individual alpha peak frequency (IAPF) and alpha bandwidth as described in Bazanova and Aftanas (2008). Namely, frequency bands showing a decrease in power of more than 20% during the eyes-open vs. eyes closed conditions in resting EEG are selected as individual alpha bands. Theta band is identified as a frequency band between 3 Hz and the lower limit of the individual alpha band, while beta band is identified as a frequency band between the upper limit of the individual alpha band and 20 Hz. Case studies have already shown that using individually adjusted bands could improve the efficiency of TBR NFT in ADHD (Kaiser, 2001; Bazanova and Aftanas, 2005, 2010).

Electromyogenic (EMG) signal resulted from forehead muscles can also explain the low efficiency of the NFT in ADHD. Several studies has shown that EEG in frequency ranges below 10 Hz and above 13 Hz are the most contaminated by EMG signal, and therefore it is almost impossible to distinguish power in theta and beta ranges from slow EMG power (Halliday et al., 1998; Goncharova et al., 2003; Chakarov et al., 2009; Shackman et al., 2009; Hashimoto et al., 2010). Although EMG contamination is greatest at the periphery of the scalp, near to the active muscles, even weak contractions can produce EMG interference that obscures or mimics the theta, mu, or beta rhythms over the entire scalp (Goncharova et al., 2003). In addition, there is also a psychological rationale to consider EMG in ADHD. Enhanced forehead muscle tone is also thought to be a sign of psychoemotional tension or mental stress (Cacioppo et al., 1988; Malmo and Malmo, 2000; Wijsman et al., 2011), and according to Braud (1978), the control over muscle tension-relaxation is impaired in ADHD, and children are not able to relax their forehead muscles (Braud, 1978; Barth et al., 2017). Indeed, a training to reduce muscle tension measured over the central forehead has been reported to be effective in the treatment of hyperactivity (Barth et al., 2017). All the above-mentioned considerations motivated the idea that the efficiency of a TBR NFT in ADHD can further increase by combining it with EMG.

In this study, we want to demonstrate the importance of individual EEG alpha activity indices such as alpha peak frequency, alpha bandwidth and alpha1/alpha2 (a1/a2) ratio, as well as that of the forehead EMG in ADHD symptomatology. We investigate whether they could serve as neurophysiological biomarkers of ADHD. Another focus of interest is whether neurofeedback training simultaneously aiming both to decrease TBR in individually determined frequency ranges and to reduce forehead muscle tension could be more efficient than conventional TBR NFT. We also hypothesize that the proposed optimized EEG/EMG NFT protocol would lead to higher alpha activity indices in addition to the steeper TBR decreases and that these effects last longer. We also hypothesize that these changes in the neurophysiology will be accompanied by corresponding behavioral improvements. Our study will demonstrate the importance of individual alpha indices and the forehead muscle tension not only in the diagnosis of ADHD but also in the optimization of the neurofeedback training protocol in treatment of ADHD.

## Materials and methods

### Participants

We recruited 94 children diagnosed with ADHD (ADHD) and 23 healthy controls (HC). All participants were male and aged between six and nine. Mean and standard deviation of age were 7.0 ± 0.20 and 7.5 ± 0.28 in the ADHD and the healthy groups, respectively. All participants' parents gave their informed consent in accordance with the Declaration of Helsinki prior to the study. The Medical Ethics Committee of the Siberian branch of the Medical Science Academy approved this study. All patients fulfilled DSM-IV criteria for ADHD (American Psychiatric Association, 2013). No children with any comorbidity were included.

ADHD group was further categorized into the three qualifying types: (a) primarily inattentive type (*n* = 29), (b) primarily hyperactive-impulsive type (*n* = 30), and (c) combined type (*n* = 35). ADHD group overall differed significantly from HC in inattention (mean_ADHD_ = 3.72, mean_HC_ = 1.10, *t* = 12.45, *p* < 0.01) and hyperactivity/impulsivity (mean_ADHD_ = 2.42, mean_HC_ = 0.91, *t* = 11.97, *p* < 0.01).

### Experimental design

First, groups were compared based on EEG, EMG, and psychometric characteristics (see section Psychometric Assessment). Then ADHD participants were randomly assigned to four groups for NFT to decrease TBR:

- standard NFT group (sNFT; *n* = 17), where standard frequency bands of 4–8 Hz (theta) and 12.5–20 Hz (beta) were used as targets (Deuschl and Eisen, 1999);
- individual neurofeedback training group (iNFT; *n* = 31), where theta and beta target frequency bands were individually adjusted (Bazanova and Aftanas, 2005, 2010);
- individual NFT group with simultaneous EMG to decrease forehead muscle tension (iNFT_EMG; *n* = 32), where individually adjusted bands and integrated power of the forehead muscle EMG were used as simultaneous targets;
- sham NFT group (*n* = 14), when participants received random audio-visual feedbacks.

NFT consisted of 10 sessions, 16 min each. Despite the considerable amount of experience in employing NFT in ADHD, the optimal amount of time (and cost) of the treatment is still on debate. A typical course of NFT involves 30–40 sessions, which is very time consuming and expensive; particularly when considering that NFT is rarely covered by health insurance. Thus, there is a constant interest in developing a NFT protocol clinically beneficial in shorter time. Hillard et al. (2013) suggests that this may be achievable. The second reason for choosing fewer sessions was the inefficiency of standard TBR NFT, which led to that parents were reluctant to prolong NFT after 10 sessions. For a fair comparison, we did not used longer training for the novel protocols either, even if they were (more) efficient. Session length of 16 min was chosen due to our experience that 6–9 years old children with ADHD could not be engaged in a computer game for longer than ca. 16 min.

**Figure 1:**
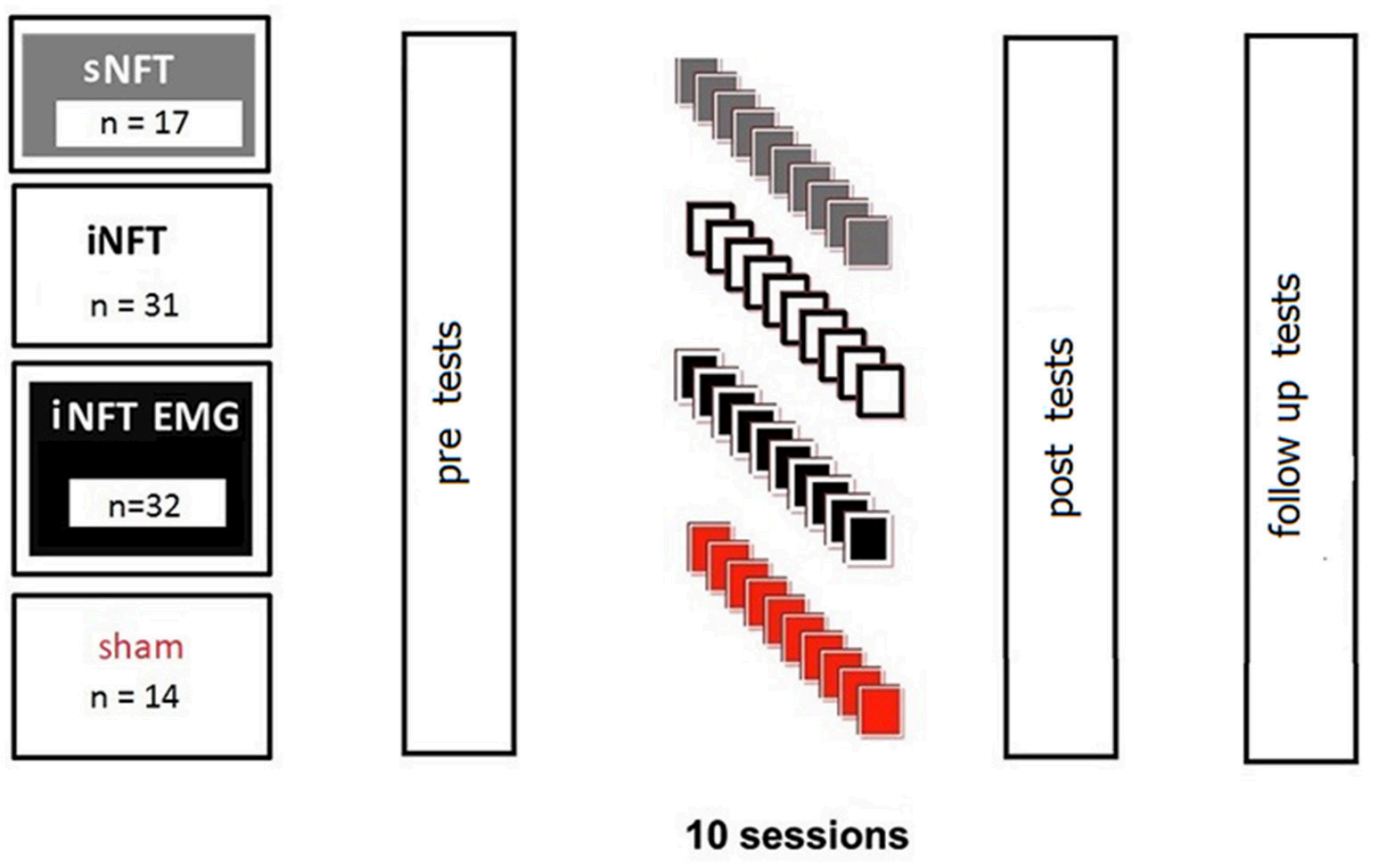
Design of the experiment.

Participants were assessed pre-, post-, and 6-month after the NFT. Assessment consisted of psychometrics, behavioral rating scales completed by parents and teachers, as well as 19-channel EEG and forehead EMG recordings (Figure 1).

### Psychometric assessment

Psychometric assessment included Go/no-Go task (Avila et al., 2004) for assessing attention, delayed gratification task (Shoda et al., 1990) for assessing impulse control and Parent and Teacher Rating Scale interview (SNAP-VI) (Swanson et al., 2013) for assessing inattention, impulsivity and hyperactivity in behavior. The tasks have been performed as follows:

- Go/no-Go task: Participants were required to make a speeded response by pressing keys “1” and “2” with the index and middle fingers of their preferred hand for letters “X” and “O,” respectively (Go). The letters were presented for 1,000 ms preceded by a 500-ms fixation point in the center of the screen. Participants were also required to inhibit responses to a Go stimulus, when it was accompanied by no-Go stimulus with a delayed onset. The no-Go stimulus was a green circle with a diameter of 3.4° presented 3.2° above the Go stimulus for 150-ms. Reaction times and number of omissions (missed stimuli) in the Go trials were measured.
- Delayed gratification task: A cake was presented to the participant, who was told to choose between two options: either eating the cake immediately (i.e., immediate reward) or waiting for another cake, so that (s)he could eat both. The experiment measured how long the participant resisted the immediate reward.

The clinical efficiency of NFT was measured as percent change in the reaction time and the number of missed stimuli in the Go-/no-Go task, as well as in the duration of delayed gratification at the post-NFT and the follow up sessions in comparison with the pre-NFT session.

### Offline EEG

We compared resting state EEG recordings of the two groups in 1 min eyes closed (EC) and 1 min eyes open (EO) condition at the pre-, post-NFT and the follow up sessions. Participants sat in a comfortable chair with a backrest and were instructed not to move their eyes during the recordings. EEG data was acquired using a Neuron-Spectrum-4 system (Neurosoft, Ivanovo, Russia) at a sampling rate of 1,000 Hz and with a bandpass filter between 0.1 and 50 Hz. The EEG electrodes were placed in accordance with the international 10–20 system: 18 electrodes were positioned in four regions: left fronto-central (LFC: Fp1, F3, C3, F7, T3,), right fronto-central (RFC: Fp2, C4, T4, F4, F8), left posterior-occipital (LPO: T5, P3, O1), and right posterior-occipital (RPO: T6, P3, O2), and signal was averaged within each area. Two reference electrodes were fixed at M1 and M2. The ground electrode was placed at Fp1 site. Impedance was kept below 5 kΩ. Data was anti-aliasing/low-pass filtered at 30 Hz.

**Figure 2:**
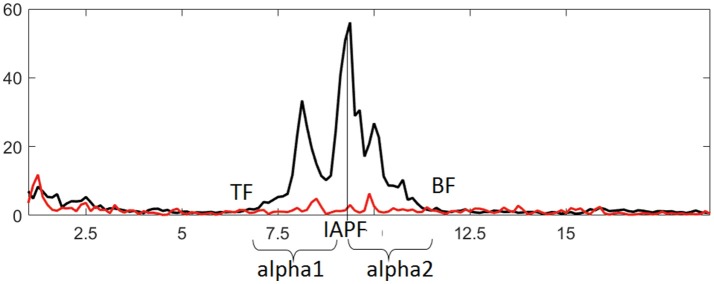
Identifying individual alpha band and derived metrics based on comparing EEG spectral power in eyes closed (black line) and eyes open (red line) conditions. IAPF, TF, and BF represent individual alpha peak frequency, theta and beta transition frequencies, respectively.

The EEG data were analyzed using WinEEG software (Mitsar Co., St. Petersburg, Russia). Eye-blink artifacts were removed using Independent Component Analysis, then the recording was divided into 4.096-s-long epochs using a Hanning window (epochs were overlapped by 50%). Only records showing an amplitude above 10 μV^2^ were included in the analysis. Analysis of EEG characteristics was carried out according to our previously published approach (Bazanova and Aftanas, 2010). Briefly, individual alpha peak frequency and alpha frequency band limits were determined at every electrode site for every participant using data from the last 30 s of EC and first 8 s of EO conditions. Frequency bands that were decreased in power by more than 20% during the EO vs. EC conditions were selected as individual alpha bands. Frequency showing the highest spectral amplitude within this band was identified as individual alpha peak frequency (IAPF). Lower and upper limits of the band [i.e., theta (TF) and beta (BF) transition frequencies] were determined where EO curve crossed the EC curve closest to IAPF. Theta and beta bands were defined between 3 Hz and TF, as well as between BF and 20 Hz, respectively. These adjusted band ranges were also used for the NFT. The alpha1 and alpha2 sub-bands were also adjusted: the alpha1 ranged from the lower limit to the individual alpha peak frequency, while the alpha2 ranged from the individual alpha peak frequency to the upper limit (Figure 2). The alpha amplitude suppression magnitude (ASM) in response to eyes open was measured as: ASM = log [(mean alpha power in EC condition – mean alpha power in EO condition) ^*^ 100%] / mean alpha power in EC condition). Alpha1 to alpha2 ratio (a1/a2) was also calculated.

### Real-time EEG and EMG

EMG and EEG data were simultaneously recorded using Bosalb system, (NIIMBB SORAMN, Novosibirsk, Russia) with the sampling rate of 720 Hz. Feedback signal was recorded from Pz scalp electrode with a bandpass filter between 3 and 30 Hz. Monopolar reference electrode was located on the right ear, and the ground electrode was located at Fp1. Electrode impedance was kept below 5 kOhm. EMG was recorded using two Ag/AgCl surface bipolar electrodes with an effective diameter of 1.6 cm placed about 3–5 cm apart on the forehead. The EMG signal was amplified and filtered with 5–350 Hz bandpass filter.

Location of Pz was selected based on the following considerations: (i) alpha activity in the parieto-occipital area is generally proposed as a modulator of the attentional states (Ergenoglu et al., 2004); (ii) alpha amplitude in posterior areas are hypothesized to reflect top-down inhibition to suppress distracting information (Min and Herrmann, 2007); (iii) posterior alpha measures are exclusively used as predictors of individual differences (Bazanova, 2011; Tenke et al., 2013; Bazanova and Vernon, 2014); (iv) test-retest investigation had shown that alpha peak frequency, bandwidth, and amplitude are the least variable and the most reproducible at the posterior brain regions (Bazanova, 2011; Bazanova and Vernon, 2014); (v) due to the nature of alpha wave generation, the changes in alpha activity recorded in posterior scalp surface reflect some generalized cerebral processes regardless of the topography (Hughes et al., 2011; Mizuhara, 2012; Kayser and Tenke, 2015); (vi) topographic analyses revealed that this electrode site is largely representative of the others while capturing the strongest effects (Kayser and Tenke, 2015).

Average power of the raw EMG was calculated over 100 ms according to the usual approach (Merletti, 1999). The resulted value was therefore the area under a power curve, measured in microvolts squared (μV^2^).

### NFT procedure

We have followed the conventional NFT protocol for ADHD to decrease TBR (Monastra, 2005). Participants underwent 10 sessions of NFT with eyes open, and each session lasted for 16 min. During training, participants sat in front of the monitor and controlled a kind of computer game by modulating their TBR. As soon as TBR dropped below the threshold, virtual flowers began to grow on the screen. Participants were instructed to find and optimize a strategy/imagery to decrease TBR, which facilitates the flower growth, as follows: “You're going to grow flowers. You can do it by letting yourself feel empty and heavy.” Immediately after the electrodes were attached, we have elaborated on the instruction to provide visual cues for imagery: “It will be easier to relax and let flowers grow up, if you just imagine yourself being a steady, heavy rock. Quiet, steady, and heavy. Relax and let your eyebrows down!” Positive verbal reinforcement was provided in every 2–3 min and the instruction with visual cue was repeated in every 5 min.

In general, thresholds were determined for each participant from 1-min pre-training baseline for each session. Reward criteria were set so that reward thresholds had to be exceeded in 70% of sampled events in a 1,000-ms period, and TBR had to range inhibit above thresholds in 30% of sampled events to receive a reward. For the sNFT group, threshold was calculated based on the mean power in standard frequency bands (4–8 Hz for theta and 12–15 Hz for beta). For the iNFT and the iNFT_EMG groups, threshold was based on mean power of individually adjusted frequency ranges (see section Offline EEG). For the iNFT_EMG group, the mean power of integrated forehead EMG was also taken into account, so that feedback signal (flower growth) appeared only when both TBR and the EMG signal lowered below the thresholds, respectively.

### Statistics

#### Psychological and neurophysiological characteristics of ADHD

Psychometrics, as well as electrophysiological (EEG, EMG) data were tested for normality by the Kolmogorov-Smirnov's test and showed normal distribution (*d* ≤ 0.033, *p* ≥ 0.2).

Psychometric and electrophysiological variables were compared pairwise between inattentive, hyperactive and combined subtypes ADHD groups, and between ADHD overall vs. HC groups using two-sample *T*-test.

For electrophysiological variables, repeated-measure 4-way ANOVA was performed using the factors SUBTYPE (3 levels: inattentive, hyperactive and combined subtypes of ADHD groups), CAUDALITY (2 levels: fronto-central and posterior-occipital), HEMISPHERE (2 levels: left and right), and CONDITION (2 levels: EC and EO). The second 3-way ANOVA with factors GROUP (2 levels: HC and ADHD overall), CAUDALITY (2 levels: fronto-central and posterior-occipital) and HEMISPHERE (2 levels: left and right) was conducted to compare overall ADHD and HC groups. ANOVAs were further extended with *post-hoc* Scheffe-test.

Pearson correlation analyses were also performed to analyze the relationship between the psychological measures and the electrophysiology. In order to identify the most powerful electrophysiological predictors of the behavior, a multiple linear regression analysis with forward stepwise selection was conducted on the data of all the 94 ADHD participants using the alpha EEG, TBR, and EMG indices as independent variables, and psychological measures (i.e., reaction time, number of missed stimuli, and duration) as dependent variables.

#### Neurofeedback training

Planned pairwise comparisons of the different NFT groups (sNFT vs. iNFT; sNFT vs. iNFT_EMG; sNFT vs. sham NFT; iNFT vs. iNFT_EMG; iNFT vs. sham NFT; iNFT_EMG vs. sham NFT) were conducted for psychometrics, behavioral measures (SNAP-VI), EMG, and feedback signals. Mixed 2-way ANOVAs were performed to evaluate the effect of within-subject factor time (3 levels: pre-NFT, post-NFT, and follow up) and between-subject factor NFT_GROUP (4 levels: sNFT, iNFT, iNFT_EMG, and sham NFT) on all variables of interest.

## Results

### Psychological and neurophysiological characteristics of ADHD

Two-sample *T*-test showed no significant difference between ADHD subtype groups in psychometrics (|*T*| ≤ 0.49, *p* ≥ 0.62) and EMG characteristics (|*T*| ≤ 0.59, *p* ≥ 0.92). All subtypes of ADHD exhibited significantly increased reaction time (*T* ≤ −3.76, *p* ≤ 0.001) and number of missed stimuli (*T* ≤ −7.91, *p* ≤ 0.001) in Go/no-go task, decreased duration (*T* ≥ 32.95, *p* ≤ 0.001) in Delayed gratification task and increased forehead muscle tension (*T* ≤ −4.38, *p* ≤ 0.001) when compared to healthy peers (Table 1).

**Table 1:** Mean and standard deviation of reaction time (in s) and number of missed stimuli in Go/no-go task, as well as duration (in s) in Delayed gratification task for the healthy and the ADHD subtype groups.

		**Healthy, *n* = 23**	**ADHD**, ***n*** = **94**		
			**Inattentive subtype, *n* = 29**	**Hyperactive subtype, *n* = 30**	**Combined subtype, *n* = 35**
Go/no-go task: reaction time in s	M±SD	0.37 ± 0.132	1.03 ± 0.209^*^	1.01 ± 0.177^*^	0.94 ± 0.223^*^
	η^2^		0.774	0.804	0.69
Go/no-go task: number of missed stimuli	M ± SD	0.30 ± 0.635	14.61 ± 6.974^*^	15.82 ± 11.576^*^	13.23 ± 6.764^*^
	η^2^		0.648	0.451	0.601
Delayed gratification task: duration in s	M ± SD	60.00 ± 0.000	21.29 ± 13.842^*^	14.48 ± 15.256^*^	16.76 ± 15.120^*^
	η^2^		0.774	0.803	0.772

* *denote significance of the differences from the healthy group at the levels of p = 0.001*.

The SUBTYPE × CAUDALITY × HEMISPHERE × CONDITION ANOVA showed no significant main effects of SUBTYPE (*F* = 0.2, *p* = 0.99, η^2^ = 0.001) and HEMISPHERE [*F* = 0.1, *p* = 0.99, η^2^ < 0.001) on alpha indices and TBR. All subtypes of ADHD showed a decreased alpha power in EO condition (i.e., significant main effect of CONDITION: *F* = 610.74, *p* < 0.001, η^2^ = 0.215). Factor CAUDALITY showed a significant main effect on alpha1 and alpha2 power, ASM, alpha1 and alpha2 bandwidth, as well as on a1/a2 ratio and TBR (*F* ≥ 20.54, *p* ≤ 0.001, η^2^ ≥ 0.012).

The GROUP × CAUDALITY × HEMISPHERE ANOVA showed significant main effect of GROUP on alpha1 and alpha2 power, ASM, alpha1 and alpha2 bandwidth, as well as on a1/a2 ratio and TBR (*F* ≥ 4.74, *p* ≤ 0.987, η^2^ ≥ 0,285). HEMISPHERE showed no significant main effect. Table 2 summarizes the *post-hoc* comparisons of neurophysiological characteristics between the ADHD subgroups and the HC group in a layout similar to Table 1. The Table 2 presents the results of ANOVA of offline EEG as extracted from the PO area.

**Table 2:** Neurophysiological characteristics of healthy and the ADHD subtype groups.

		**Healthy, *n* = 23**	**ADHD**, ***n*** = **94**		
			**Inattentive subtype, *n* = 29**	**Hyperactive subtype, *n* = 30**	**Combined subtype, *n* = 35**
Individual alpha peak frequency. Hz	M ± SD	9.32 ± 0.685	8.42 ± 0.292^*^	8.58 ± 0.508^*^	8.49 ± 0.405^*^
	η^2^		0.566	0.285	0.376
A1/a2 ratio	M ± SD	1.04 ± 0.053	1.82 ± 0.622^*^	2.11 ± 0.076^*^	1.57 ± 0.519^*^
	η^2^		0.409	0.475	0.302
Individual alpha2 band width. Hz	M ± SD	2.36 ± 0.346	1.38 ± 0.413^*^	0.984 ± 0.344^*^	1.35 ± 0.466^*^
	η^2^		0.622	0.804	0.587
Alpha suppression. log%	M ± SD	3.53 ± 0.514	1.45 ± 0.311^*^	1.57 ± 0.341^*^	1.54 ± 0.361^*^
	η^2^		0.868	0.844	0.843
Theta/beta ratio	M ± SD	0.66 ± 0.210	6.61 ± 2.185^*^	10.17 ± 3.770^*^	7.44 ± 3.491^*^
	η^2^		0.763	0.743	0.609
EMG	M ± SD	5.86 ± 1.486	8.64 ± 3.017^*^	8.62 ± 2.455^*^	9.14 ± 3.750^*^
	η^2^		0.240	0.308	0.223

*Offline PO EEG and EMG data of healthy and the ADHD subtype groups before the training*.

* *denote significance of the differences from the healthy group at the levels of p = 0.001*.

Table 2 Neurophysiological characteristics of healthy and the ADHD subtype groups. Offline PO EEG and EMG data of healthy and the ADHD subtype groups before the training.

Pearson correlation analysis showed significant relationship between Go/no-Go test results and EEG indices: Both reaction time and number of missed stimuli correlated negatively with individual alpha peak frequency (*r* ≤ −0.67, *p* < 0.05), ASM (*r* ≤ −0.48, *p* < 0.05), alpha bandwidth (*r* ≤ −0.40, *p* < 0.05), and positively with a1/a2 ratio (*r* ≥ 0.64, *p* < 0.05), and TBR (*r* ≥ 0.58, *p* < 0.05). Duration of the Delayed gratification task correlated positively with individual alpha peak frequency (*r* = 0.66, *p* < 0.05), ASM (*r* = 0.85, *p* < 0.05), alpha bandwidth (*r* = 0.60, *p* < 0.05), and negatively with a1/a2 ratio (*r* = −0.60, *p* < 0.05) and TBR (*r* = −0.74, *p* < 0.05). Muscle tension measured with EMG also correlated positively with reaction time (*r* = 0.38, *p* < 0.05) and missed stimuli number (*r* = 0.19, *p* < 0.05) in Go/no-go task, and negatively with the duration in Delayed gratification task (*r* = −0.40, *p* < 0.05). According to the multiple linear regression analysis, where pre-training psychological measures of ADHD were included as dependent variables and all EEG and EMG variables were included as predictors, individual alpha peak frequency, individual alpha2 bandwidth, a1/a2 ratio predicted all three psychological measures of ADHD significantly (|beta| ≥ 0.151, |*r*_semipartial_| ≥ 0.081, *p* ≤ 0.036) (Table 3, typed in bold).

**Table 3:** Multiple stepwise linear regression analysis of the predictive power electrophysiological metrics when considering reaction time (in s), number of missed stimuli in Go/no-Go test and duration (in s) in Delayed gratification task.

**Dependent Variables**	**Predictors**	**Beta**	***r*_partial_**	***r*_semipartial_**	**Adjusted *r***	***F***	***df***	***p***
Go/no-go task: reaction time in s	**Individual alpha peak frequency in Hz**	−**0.250**	−**0.389**	−**0.194**	0.775	58.171	7.109	**0.000**
	*Individual alpha bandwidth in Hz*	−*0.147*	−*0.202*	−*0.095*				*0.034*
	**Individual alpha2 bandwidth in Hz**	−**0.212**	−**0.200**	−**0.094**				**0.036**
	**a1/a2 ratio**	**0.250**	**0.351**	**0.172**				**0.000**
	*ASM*	−*0.166*	−*0.244*	−*0.116*				*0.010*
	TBR	0.108	0.138	0.064				0.148
	EMG	0.051	0.098	0.045				0.307
Go/no–go task: number of missed stimuli	**Individual alpha peak frequency in Hz**	−**0.376**	−**0.494**	−**0.291**	0.720	43.374	7.109	**0.000**
	Individual alpha bandwidth in Hz	−0.025	−0.032	−0.016				0.741
	**Individual alpha2 bandwidth in Hz**	−**0.580**	−**0.448**	−**0.257**				**0.000**
	**a1/a2 ratio**	**0.254**	**0.323**	**0.175**				**0.001**
	*ASM*	*0.127*	*0.170*	*0.089*				*0.074*
	TBR	−0.066	−0.077	−0.039				0.425
	*EMG*	−*0.140*	−*0.234*	−*0.124*				*0.013*
Delayed gratification task: duration in s	**Individual alpha peak frequency in Hz**	**0.151**	**0.341**	**0.117**	0.889	134.410	7.109	**0.000**
	Individual alpha bandwidth in Hz	0.077	0.152	0.049				0.112
	**Individual alpha2 bandwidth in Hz**	**0.183**	**0.244**	**0.081**				**0.010**
	**a1/a2 ratio**	−**0.159**	−**0.321**	−**0.109**				**0.001**
	*ASM*	*0.446*	*0.694*	*0.311*				*0.000*
	*TBR*	−*0.143*	−*0.256*	−*0.085*				*0.007*
	EMG	−0.051	−0.138	−0.045				0.147

### Neurofeedback training

EEG and EMG characteristics are based on the offline EEG and EMG data. At the pre-training session, psychometric, EEG and EMG characteristics showed no difference between the NFT groups (*T* ≤ 1.55, *p* ≥ 0.122).

**Figure 3:**
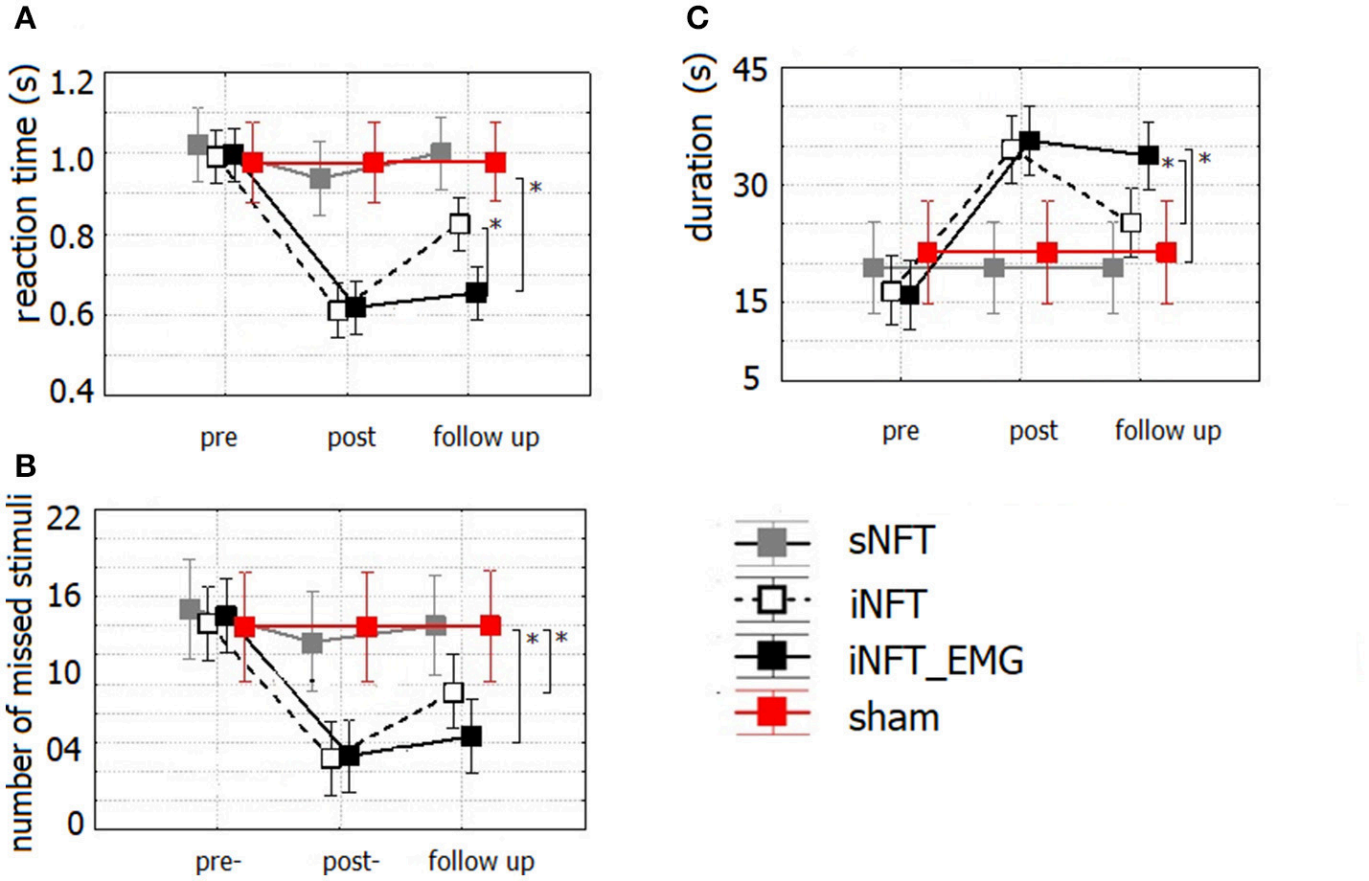
Mean and standard deviation of **(A)** reaction time (in s) and **(B)** number of missed stimuli in Go/no-Go task, as well as of **(C)** duration (in s) in Delayed gratification task at the pre-NFT, post-NFT session and six month after the NFT. ^*^Means that the group difference is significant (*p* < 0.005).

**Figure 4:**
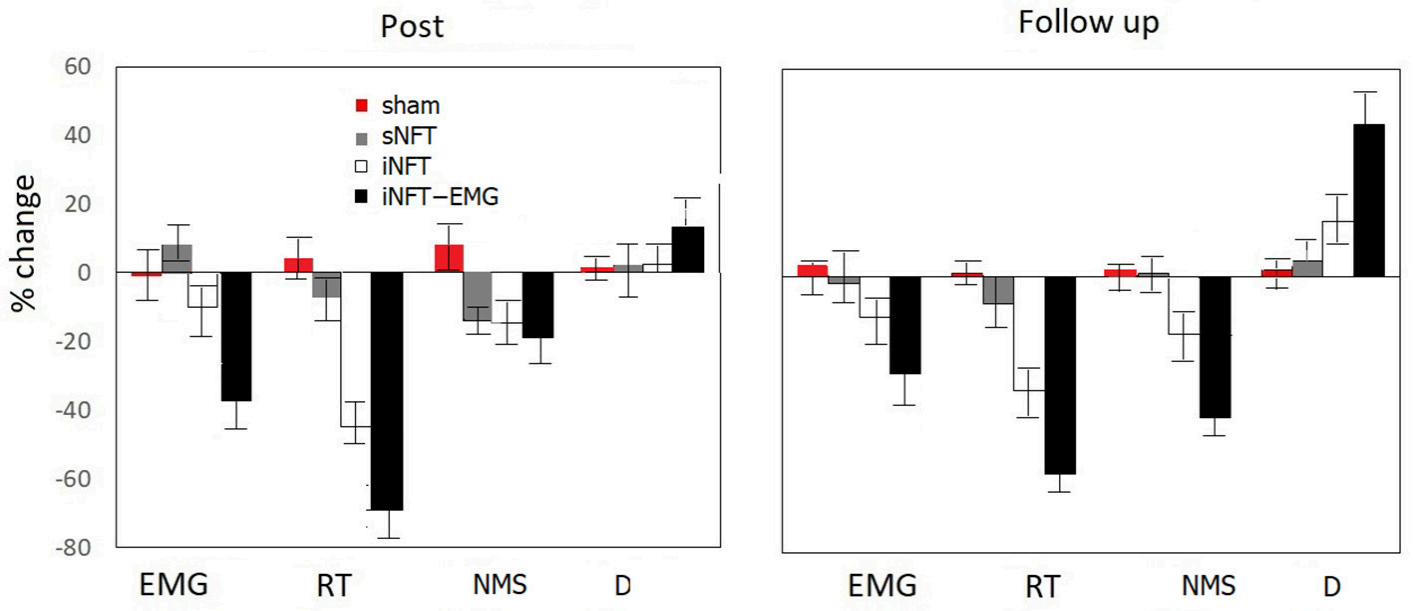
Mean and standard deviation of percentage change in EMG, reaction time (RT) and numbers of missed stimuli (NMS) in Go/no-Go task, as well as in duration of Delayed gratification task (D) at post-NFT session and six month after the NFT relative to the pre-NFT session.

Mixed ANOVA on psychometric outcomes revealed significant main effect of TIME (*F* = 5.04, *p* < 0.001, η^2^ = 0.196) and significant TIME × NFT_GROUP interaction on all studied variables (*F* = 2.26, *p* < 0.001, η^2^ = 0.116) (Figure 3). *Post-hoc* tests at the post-training session revealed significant differences between the groups in psychological measures: iNFT and iNFT_EMG groups showed decreased reaction time and number of missed stimuli in Go/no-go task, as well as increased duration of Delayed gratification task in comparison with both sNFT and sham NFT groups (*T* ≥ 8.84, *p* ≤ 0.001, η^2^ ≥ 0.266). All these effects remained until the follow-up session 6 months after the NFT in the iNFT_EMG group only (*T* = 5.30, *p* ≤ 0.001, η^2^ ≥ 0.182). iNFT group showed improvement only in the number of missed stimuli (Figure 3). NFT efficiency measured with psychological measures was the highest in the iNFT_EMG group (η^2^ > 0.46), while neurofeedback training effect severely diminished in other groups by the time of the follow-up session (Figure 4).

**Figure 5:**
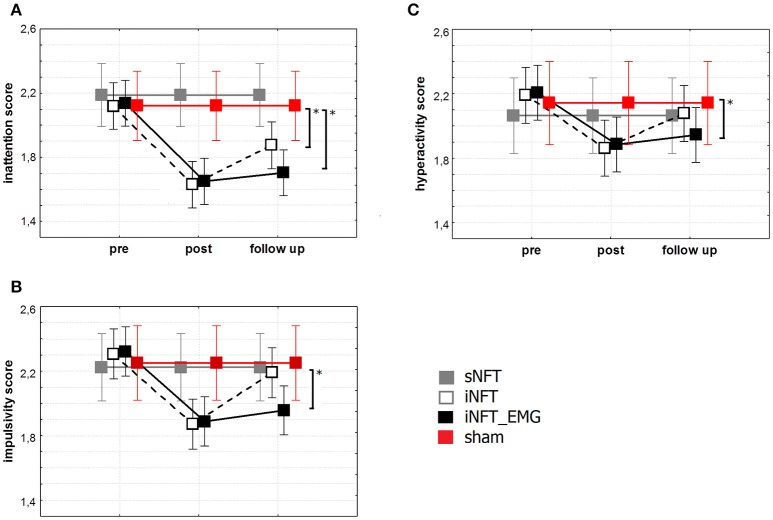
Mean and standard deviation of SNAP-VI **(A)** inattention, **(B)** impulsivity and **(C)** hyperactivity scores at the pre-NFT, post-NFT session, and six month after the NFT. ^*^Means that the group difference is significant (*p* < 0.005).

Mixed ANOVA on behavior assessment with Parent and Teacher Rating Scale interview (SNAP-VI) showed significant main effect of NFT_GROUP (*F* ≥ 4.627, *p* < 0.001, η^2^ ≥ 0.060) and TIME (*F* ≥ 2.651, *p* < 0.01, η^2^ ≥ 0.028) on all variables (Figure 5). *Post-hoc* tests at the post-training session revealed significant differences between the groups: iNFT and iNFT_EMG groups showed a significant decrease in inattention and impulsivity scores in comparison with both sNFT and sham NFT groups (|*T*| ≥ 3.239, *p* ≤ 0.001, η^2^ ≥ 0.060). NFT efficiency was the highest in the iNFT_EMG group when considering inattention (*F* = 7.703, *p* < 0.001, η^2^ > 0.060) and impulsivity scores (*F* = 3.887, *p* < 0.001, η^2^ > 0.600).

**Figure 6:**
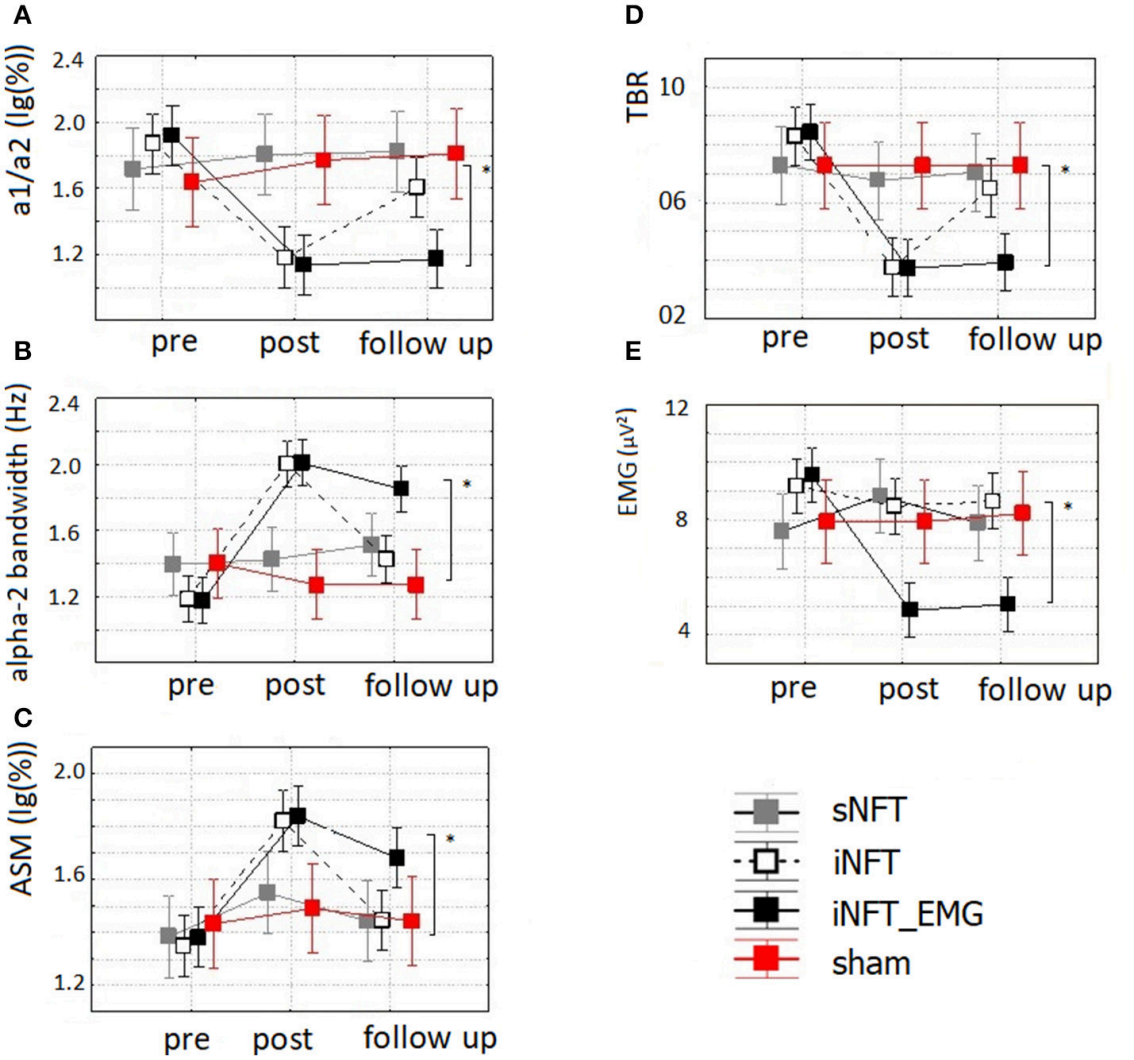
Mean and standard deviation of **(A)** alpha-1/alpha-2 ratio (a1/a2), **(B)** individual alpha-2 bandwidth (alpha-2 bandwidth) (in Hz), **(C)** alpha amplitude suppression magnitude (ASM) (in log-ratio), **(D)** theta/beta ratio (TBR) and **(E)** EMG of frontal muscles (in μV^2^) at the pre-NFT, post-NFT session, and six month after the NFT. ^*^Means that the group difference is significant (*p* < 0.005).

Mixed ANOVA on EEG indices also revealed significant main effect of TIME (*F* ≥ 4.42, *p* < 0.001, η^2^ ≥ 0.131) and significant TIME × NFT_GROUP interaction on alpha activity indices and TBR (*F* ≥ 2.40, *p* < 0.001, η^2^ ≥ 0.102) (Figure 6). *Post-hoc* tests at the post-training session revealed significant differences between the groups only in ASM, alpha2 bandwidth, a1/a2 ratio and TBR: iNFT and iNFT_EMG groups showed increased ASM, alpha2 band width, as well as decreased a1/a2 ratio and TBR in comparison with both sNFT and sham NFT groups (*T* ≥ 5.11, *p* < 0.001, η^2^ ≥ 0.365). All these effects were present at the follow-up session only for the iNFT_EMG group (*T* ≥ 3.29, *p* ≤ 0.001, η^2^ ≥ 0.298).

Mixed ANOVA on muscle tension as measured with EMG showed significant main effect of TIME (*F* = 4.09, *p* < 0.05, η^2^ = 0.026) and TIME × NFT_GROUP interaction (*F* = 7.00, *p* < 0.001, η^2^ = 0.134) (Figure 6). *Post-hoc* tests at the post-training session revealed that iNFT_EMG group exhibited muscle tension significantly lower than the other groups (*T* = 13.40, *p* < 0.001, η^2^ = 0.308), and this effect were also present at the follow-up session (*T* = 13.58, *p* < 0.001, η^2^ = 0.311) (Figure 4).

## Discussion

Individual variances of the EEG profile may confound the neurofeedback training, if ignored. Previous electroencephalographic studies attribute persistent increase in slow wave activity during resting state mostly to increase in theta power in ADHD (Chabot and Serfontein, 1996; Hermens et al., 2005). These studies consider elevated theta power and TBR according to standard band limits as the strongest biomarkers of ADHD. However, recent studies have failed to replicate their findings (Loo et al., 2009, 2013; van Dongen-Boomsma et al., 2010; Liechti et al., 2013). One possible reason for this disagreement could be that the standard approach to estimate TBR may not fit for all due to individual variability. A more thorough assessment of the individual EEG profile is necessary to identify and investigate the role of electrophysiological biomarkers and to adjust NFT protocols accordingly. General recognition that physicians need to take individual variability into account is driving huge interest in “precision” medicine (Schork, 2015). In NFT literature, individualization usually implemented by comparing one's EEG profile to age-regressed equations for normal individuals. See Lansbergen et al. (2011) as an example. However, EEG profiles are still determined based on standard frequency ranges, which can explain the low clinical efficiency (electrophysiology was not reported). The present study emphasize the importance of individualized adjustment of alpha activity measures when evaluating the electrophysiological status of the patients prior to NFT. However, it also requires acquiring EEG data during eyes closed condition, which most studies prefer not to do to avoid participants from becoming drowsy. Therefore, they cannot adjust band limits individually based on alpha amplitude suppression.

Our study replicated most of the previous findings regarding the electrophysiological profile of ADHD. We have found decreased alpha and beta powers in agreement with the literature (Bresnahan and Barry, 2002; Hobbs et al., 2007; Poil et al., 2014; Giertuga et al., 2017). We could also demonstrate the left shift of individual alpha peak frequency in ADHD in comparison with HC (Bazanova and Aftanas, 2010; Arns et al., 2012; Arns, 2013). We have also proved that alpha band characteristics measured at posterior areas could predict individual differences between ADHD and HC (Bazanova, 2011; Tenke et al., 2013; Bazanova and Vernon, 2014). According to Min and Herrmann (2007), decrease in alpha power and bandwidth at posterior areas reflect deficit in top-down inhibition to suppress distracting information in ADHD children. In addition to previous findings that alpha activity is altered in ADHD, we have also found that a1/a2 ratio, alpha peak frequency and alpha2 bandwidth are the most powerful predictors of inattention, impulsivity and hyperactivity. Our results showed that decrease in individually adjusted alpha indices, such as alpha peak frequency, alpha2 bandwidth, ASM, and a1/a2 ratio could predict at least 40% of the decrease in duration of Delayed gratification task and reaction time in Go/no-go task, as well as of the increase in number of missed stimuli in Go/no-Go task. More importantly, (individualized) alpha activity metrics have been proved to be more powerful predictors of ADHD symptoms than TBR. This is further supported by the fact that the groups, which received individualized TBR NFT showed significant improvement in symptoms in parallel with corresponding changes in (individualized) alpha activity metrics. Further implication of these findings is that the accurate identification of the alpha sub-bands allows for a NFT specifically target these alpha activity metrics; for example to decrease the alpha-1/alpha-2 ratio directly, which might be even more efficient in reducing ADHD symptoms.

Hyperactivity is also characterized by redundant motoric elements; i.e., patient perform more movements than necessary for a given motor task. This contextual redundancy reflects the inefficiency of inhibitory processes in the sensorimotor coordination (Sterrnan and Friar, 1972; Bernstein, 1996). Previous studies of a sensorimotor rhythm localized at the sensorimotor cortex in cats indicated its functional relationship with thalamo-cortical inhibitory discharge (Howe and Strerman, 1972) and voluntary suppression of movements (Wyrwicka and Sterman, 1968). Over the last decade, a growing number of report also suggest the important role of the alpha oscillations in top-down mechanism of neural inhibition (see Pfurtscheller and Lopes da Silva, 1999; Klimesch et al., 2007; Klimesch, 2012) and thus in cognitive task performance. For further proof of this top-down mechanism, recent studies has demonstrated that inhibition of redundant muscle activity was accompanied by decrease in EMG power and a simultaneous increase in EEG upper alpha and SMR power (Sammler et al., 2007; Bazanova et al., 2009; Bowers et al., 2014). In contrast, an increase in forehead muscle tension was shown to be accompanied by decrease in EEG alpha power (Barry et al., 2007; Bazanova and Vernon, 2014). The close relationship between the EEG alpha and EMG powers in controlling task-relevant neural activity and mental stress implies that the role of EEG upper alpha activity in inhibiting redundant motor behavior and reducing the excessive psychoemotional tension. Therefore, it is not surprising that individually adjusted alpha indices and EMG can predict ADHD symptoms; which gives an additional rationale to adjust TBR according to individually adjusted alpha band limits in NFT (Bazanova and Aftanas, 2010).

Sensorimotor integration is a key for optimal learning, and increase in alpha-2 activity with a simultaneous decrease in forehead EMG is proved to be the most reliable markers of the efficient learning (Sammler et al., 2007; Bazanova et al., 2009; Bowers et al., 2014). These results are in concordance with that we found stronger effect in both the electrophysiological measures and the ADHD symptoms at the 6-months follow-up only for the iNFT_EMG group. These results prove that individualized TBR NFT was significantly more efficient in improving attention and impulse control in children with ADHD than standard NFT protocol, and that its effect takes longer when combined with simultaneous EMG.

## Conclusion

By means of group comparison followed by regression analysis, we identified individualized alpha peak frequency, alpha1/alpha2 ratio and forehead muscle tension as the most powerful predictors of ADHD symptoms.

We confirmed the lack of efficiency of TBR NFT when using standard bands, as well as its efficiency when EEG bands has been individually adjusted. We further demonstrated that combining NFT with muscle tension control training could lead to a longer lasting training effect. These research findings supports our claim that efficiency of NFT can be improved by considering individual characteristics of the EEG and the muscle tension.

## Author contributions

OB had substantial contributions to the conception of the work, analysis, interpretation of data for the work and revising it critically for important intellectual content and final approval of the version to be published. She provided of agreement to be accountable for all aspects of the work in ensuring that questions related to the accuracy or integrity of any part of the work are appropriately investigated and resolved. TA had substantial contributions to the analysis, interpretation of data for the work and revising it critically for important intellectual content and final approval of the version to be published. ES had a contributions to the design, acquisition, analysis and interpretation of data for the work. She was drafting the work and revising it critically for important intellectual content.

### Conflict of interest statement

The authors declare that the research was conducted in the absence of any commercial or financial relationships that could be construed as a potential conflict of interest.
